# Leptin Therapy Improves Metabolic Dysfunction in Immune Checkpoint Inhibitor–induced Lipodystrophy

**DOI:** 10.1210/jcemcr/luaf340

**Published:** 2026-02-16

**Authors:** Mona Vahidi Rad, Suhail Saad-Omer, Bharath Nair Suresh Kumar Bindu, Vinaya Simha

**Affiliations:** Department of Endocrinology, Mayo Clinic, Rochester, MN 55905, USA; Department of Endocrinology, Mayo Clinic, Rochester, MN 55905, USA; Department of Endocrinology, Mayo Clinic, Rochester, MN 55905, USA; Department of Endocrinology, Mayo Clinic, Rochester, MN 55905, USA

**Keywords:** generalized lipodystrophy, leptin, immune checkpoint inhibitor

## Abstract

Immune checkpoint inhibitors (ICI) have transformed cancer therapy but can induce a spectrum of endocrine complications. Acquired generalized lipodystrophy (AGL) is a rare adverse effect, leading to severe metabolic derangements including insulin-resistant diabetes, dyslipidemia, and hepatic steatosis. Marked hypoleptinemia is noted in these patients, and leptin-replacement therapy is known to be beneficial in patients with other forms of AGL. Though rare, T-cell lymphoma has been reported in patients with AGL on leptin therapy, and there has been much debate on the association between hyperleptinemia and cancer. The safety and efficacy of leptin therapy in patients with ICI-induced AGL and cancer are therefore not clear. We report a 66-year-old woman with metastatic lung adenocarcinoma who developed multiple endocrine disorders including AGL with metabolic abnormalities following pembrolizumab therapy. Initiation of daily metreleptin led to marked improvement in dyslipidemia, glycemic control, and hepatic steatosis, with no evidence of cancer recurrence after 2 years of leptin-replacement therapy. While further studies are warranted to establish the long-term efficacy and safety of leptin in ICI-induced AGL patients with a history of malignancy, this case report provides useful information for the management of these rare, challenging patients.

## Introduction

Endocrine adverse effects associated with immune checkpoint inhibitors (ICI) therapy encompass a range of conditions, including hypopituitarism due to hypophysitis, thyroid dysfunction (primary or secondary), adrenal insufficiency (primary or secondary), type 1 diabetes mellitus, and, more infrequently, hypoparathyroidism. In addition, acquired generalized lipodystrophy (AGL) due to autoimmune adipose tissue inflammation is a rare complication that has been recently reported [1, 2]. It is characterized by subclinical panniculitis; generalized fat loss; and impaired adipocytokine secretion leading to metabolic abnormalities such as hyperglycemia, hyperlipidemia, and steatohepatitis [3]. In general, AGL may be associated with panniculitis (approximately 25%) or autoimmune disorders (approximately 25%), or it may occur without a known cause (idiopathic, 50%) [3], and ICI-induced AGL may represent a novel subtype.

Leptin replacement therapy has been demonstrated to improve metabolic abnormalities in patients with generalized lipodystrophy, whether congenital or acquired, and is an approved treatment option for these individuals [3, 4]. However, there is a need for caution in initiating leptin replacement therapy in patients with ICI-induced AGL who have a known malignancy. Studies have reported leptin as having both potentially detrimental and beneficial roles in cancer [5]. A previous case series had also reported T-cell lymphoma in 3 patients with AGL who were on leptin replacement therapy [6]. But considering the limited therapeutic options for generalized lipodystrophy and its association with metabolic complications, it is important to assess the safety and efficacy of leptin therapy in ICI-induced AGL.

We previously presented a patient with a history of metastatic lung adenocarcinoma and ICI-induced AGL who subsequently developed metabolic complications, including insulin-requiring diabetes mellitus, dyslipidemia, and hepatic steatosis [2]. We now report an update on this patient, who was treated with leptin replacement therapy for approximately 2 years, leading to resolution of the metabolic abnormalities and no evidence of cancer recurrence. While leptin has demonstrated efficacy in other forms of generalized lipodystrophy, it has not been studied in ICI-induced AGL. This represents the first reported use of leptin in a patient with cancer and ICI-induced AGL, underscoring the potential safety and effectiveness of this treatment in managing metabolic complications in such cases.

## Case Presentation

We previously reported [2] a 66-year-old woman with metastatic lung adenocarcinoma treated with pembrolizumab who developed multiple immune-related endocrine complications, including primary hypothyroidism, secondary adrenal insufficiency, and new-onset insulin-requiring diabetes without autoantibodies approximately 10 to 15 months into therapy [2]. As described previously, she experienced a significant 20 kg weight loss and developed generalized fat loss, starting with the face and then involving the entire body. The prominence of limb muscles and subcutaneous veins along with hepatomegaly led to suspicion for AGL.

## Diagnostic Assessment

In addition to new-onset diabetes mellitus, the patient also developed new metabolic abnormalities, including hypertriglyceridemia ranging from 234 to 578 mg/dL (SI: 2.64-6.53 mmol/L) (reference range, <150 mg/dL [SI: <1.7 mmol/L]) and elevated liver enzymes (3-4 times the upper limit of normal). Subsequent liver biopsy revealed severe macrovesicular steatosis and mild focal lobular inflammation. Notably, her serum leptin concentration measured during the initial assessment was undetectable at <0.6 ng/mL (reference range, 3.3-18.3 ng/mL) despite preserved fat in the trunk and limbs at this time.

## Treatment

She was initiated on insulin therapy for new-onset diabetes and advised to follow a strict low-fat diet (less than 10-20 grams fat per day) with initial improvement in glycemic control and hyperlipidemia. Unfortunately, despite improvements in metabolic function, the patient continued to have weight loss predominantly in the facial area, which then became generalized and was associated with a progressive increase in insulin requirement up to 2 U/kg. Pioglitazone 30 mg daily was trialed without any beneficial effect on fat loss and was ultimately discontinued after 4 weeks due to pedal edema. During this time, she also noted increased hunger and was started on glucagon-like peptide 1 receptor agonist (GLP-1 RA) therapy, initially dulaglutide 0.75 mg weekly and then changed to semaglutide 3 mg orally daily when she did not tolerate dulaglutide. She has remained on this low dose as she could not tolerate higher doses, but it did help improve glycemic control along with insulin therapy. Despite adequate glycemic control, the patient was noted to have persistent hypertriglyceridemia and worsening hepatic steatosis as documented on magnetic resonance imaging liver elastography (mean fat fraction of 18%). Given these findings, the pros and cons of leptin replacement were discussed with the patient, who opted to start leptin therapy. The patient started 5 mg of metreleptin (human recombinant leptin) by subcutaneous injection daily about 4 months after she had started glucagon-like peptide 1 receptor agonist therapy. Labs prior to starting leptin replacement revealed hypertriglyceridemia with triglycerides of 578 mg/dL (SI: 6.53 mmol/L), hemoglobin A1c of 6.7% (SI: 50 mmol/mol), mild transaminitis with alanine aminotransferase of 49 (SI: 0.82 μkat/L) (reference range, 7-45 U/L [SI: 0.12-0.75 μkat/L]), and thrombocytopenia with platelet count of 63 × 10³/μL (reference range, 157-371 × 10³/μL).

## Outcome and Follow-up

The patient was reevaluated 1 month after starting metreleptin and reported improved energy levels and decreased appetite but was unsure if this was due to low-dose oral semaglutide or leptin therapy. Insulin requirements were also found to have decreased. There was progressive improvement in glucose and lipid levels and by 8 months of leptin-replacement therapy, insulin therapy was completely discontinued. Repeat labs 1 year after starting leptin therapy showed a significant improvement in triglyceride levels to 71 mg/dL (SI: 0.8 mmol/L), resolution of transaminitis, and hemoglobin A1c of 4.0% (SI: 20 mmol/mol). Repeat magnetic resonance imaging liver elastography after 2 years of therapy revealed improvement in hepatosteatosis (Table 1). The patient continues to tolerate metreleptin without any complications and has remained on the 5 mg daily dose along with 3 mg oral semaglutide. She has been undergoing regular cancer surveillance every 6 months with chest and abdomen computed tomography scans and whole-body 18-fluorodeoxyglucose positron emission tomography scans. Two years after starting leptin therapy, she has not had any documented recurrence of previous lung adenocarcinoma or discovery of any new malignancies.

**Table 1.** Laboratory, imaging findings, and patient's medications before and after leptin therapy, showing improvements in metabolic parameters and hepatic steatosis

Laboratory/imaging test	Before Leptin therapy	After 1 year of leptin therapy	Reference range
Triglycerides	578 mg/dL (SI: 6.53 mmol/L)	71 mg/dL (SI: 0.8 mmol/L)	<150 mg/dL (SI: <1.7 mmol/L)
Hemoglobin A1c	6.7% (SI: 50 mmol/mol)	4.0% (SI: 20 mmol/mol)	<6.5% (SI: 48 mmol/mol)
ALT	49 U/L (SI: 0.82 μkat/L)	37 U/L (SI: 0.62 μkat/L)	7-45 U/L (SI: 0.12-0.75 μkat/L)
AST	35 U/L (SI: 0.58 μkat/L)	37 U/L (SI: 0.62 μkat/L)	8-43 U/L (SI: 0.13-0.72 μkat/L)
Platelet count	63 × 10³/µL	54 × 10³/µL	157-351 × 10³/µL
Liver fat fraction (MRI)	18%	0%	(No established reference)
Medications	Insulin (2 U/kg), metformin 2 g/day, semaglutide 3 mg/day oral, tenofibrate 145 mg/day	Metreleptin 5 mg/day, metformin 500 mg/day, semaglutide 3 mg/day oral	

Abbreviations: ALT, alanine aminotransferase; AST, aspartate aminotransferase; MRI, magnetic resonance imaging.

## Discussion

We report a patient with a rare presentation of AGL with severe metabolic complications following ICI therapy for metastatic lung adenocarcinoma who was treated with human recombinant leptin resulting in remarkable metabolic improvement. There was a dramatic improvement in hyperglycemia and hyperlipidemia and resolution of hepatic steatosis, facilitating discontinuation of insulin and fenofibrate therapy and underscoring leptin's potential role in managing immune-related lipodystrophy. Leptin is an adipocyte-derived hormone that regulates energy homeostasis, appetite, and body weight but also plays a central role in the pathogenesis of metabolic complications [7]. Previous case reports and studies have demonstrated the use of metreleptin in both pediatric and adult patients with AGL showing significant improvement in metabolic parameters such as reductions in hemoglobin A1c and triglycerides and, in some cases, improved hepatic and autoimmune disease activity [8, 9]. However, such reports largely precede the advent of ICI-induced lipodystrophy, and they involve patients with other forms of generalized lipodystrophy.

Despite promising experience with leptin in AGL, there was initial apprehension about introducing leptin replacement in this patient, given the underlying or recent history of cancer. The association between hyperleptinemia and cancer risk remains controversial. Some studies suggest that leptin may play a protumoral role, with elevated leptin levels linked to increased tumor growth. It has been reported to promote cell proliferation in breast, esophageal, and prostate cancers and to potentially be associated with colorectal cancer, endometrial cancer, and hepatocellular carcinoma [5]. There are several interrelated mechanisms by which hyperleptinemia is postulated to increase the risk for cancer. First, leptin activates oncogenic signaling pathways to drive cell proliferation and survival in cancers such as endometrial cancer and breast cancer [5]. Leptin was also found to disrupt epithelial cell polarity and promote epithelial-mesenchymal transition, a process critical for tumor invasion and metastasis [10]. Additionally, hyperleptinemia induces a chronic proinflammatory state, promoting a tumor-supportive microenvironment by inducing proinflammatory cytokine production and facilitating immune cell polarization that favors tumor progression [11].

There is also concern about lymphoma development and progression in patients with AGL on metreleptin therapy. During the initial clinical development of leptin replacement therapy, 3 patients with AGL were noted to develop T-cell lymphoma [6]; however, a more recent systematic analysis revealed a greater percentage of patients with AGL who developed lymphoma were metreleptin-naïve compared to those on leptin therapy, suggesting that lymphoma occurrence is more likely related to the disease's underlying pathophysiology than to the pharmacological effects of metreleptin [12].

Conversely, leptin has been demonstrated to exhibit antitumoral effects in human pancreatic cancer cell lines [5]. Some studies have also suggested that obesity may play a paradoxically favorable role in tumorigenesis and has been associated with improved outcomes in certain cancers [13]. The term *leptin paradox* has been used to explain the dual role of leptin in cancer, referring to its potentially both detrimental and beneficial effects in tumor development [5].

We present the first case of leptin therapy in a patient with ICI-induced AGL. Despite the reported association between leptin therapy and cancer risk, our patient responded well to leptin therapy with improvement in her metabolic complications and no documented recurrence of her malignancy or development of any new malignancies. While further studies are required to document safety and evaluate the role of leptin therapy in patients with ICI-induced lipodystrophy, it is not necessary to withhold leptin therapy in these patients for theoretical concerns about promoting recurrence of underlying malignancy.

## Learning Points

- AGL can arise as a rare endocrine complication of ICI therapy.
- AGL is associated with several metabolic complications, including insulin-resistant diabetes, hypertriglyceridemia, and hepatic steatosis.
- Leptin replacement therapy can significantly improve metabolic complications in ICI-induced AGL without provoking cancer recurrence, emphasizing its therapeutic potential, though further studies are needed to evaluate long-term safety.

## Contributors

All authors made individual contributions to authorship. M.V.R., S.S., B.N.S.K.B., and VS were involved in patient management and manuscript submission. All authors reviewed and approved the final draft.

## Data Availability

Some or all datasets generated during and/or analyzed during the current study are not publicly available but are available from the corresponding author on reasonable request.
